# The effects of upper airway obstruction on oxygen consumption and ventilatory variables in Colombian criollo horses

**DOI:** 10.1093/jvimsj/aalag111

**Published:** 2026-06-17

**Authors:** Shannon L Massie, Angélica María Zuluaga-Cabrera, Warwick M Bayly, Lady Consuelo Calixto Vega, Viviana Elena Castillo Vanegas, Renaud Léguillette

**Affiliations:** Faculty of Veterinary Medicine, University of Calgary, Calgary T2N 1N4, Canada; GISCA Research Group, Institución Universitaria Visión de las Américas, 050031, Laureles, Medellín, Colombia; Department of Veterinary Clinical Sciences, College of Veterinary Medicine, Washington State University, Pullman, WA 99164, United States; Universidad de La Salle, 110231, Chapinero, Bogotá, Colombia; Vitalab diagnostico Veterinario, Rionegro, Antioquia-colombia GISCA research group, Institución Universitaria Visión de las Américas, 054048, Llanogrande, Rionegro, Colombia; Faculty of Veterinary Medicine, University of Calgary, Calgary T2N 1N4, Canada

**Keywords:** pharyngeal collapse, aerobic capacity, exercise induced pulmonary hemorrhage

## Abstract

**Background:**

While conditions associated with upper airway obstruction (UAO) are reported in Colombian Criollo horses, their effect on ventilation and performance is unknown.

**Hypothesis/Objectives:**

To investigate how oxygen consumption ($\dot{\rm V}$O_2_), ventilation,, and exercise-induced pulmonary hemorrhage (EIPH) are affected by UAO in horses performing at natural gaits.

**Animals:**

Twenty-three client-owned Colombian Criollo horses.

**Methods:**

A cross-sectional observational study assigned horses to a control (C), or UAO group after overground endoscopy. All horses performed a standardized submaximal exercise test while wearing an ergospirometry facemask. Post-exercise blood lactate concentration and bronchoalveolar lavage cytological results were assessed.

**Results:**

Twelve horses had UAO, with 6/12 having multiple diagnoses. Recurrent laryngeal neuropathy was most prevalent (83%), followed by nasopharyngeal collapse (42%). Mean $\dot{\rm V}$O_2_ and minute ventilation were not different between C (79.4 ± 22.0 mL/(kg min); 723 ± 203 L/min) and UAO (88.2 ± 23.2 mL/(kg min); 648 ± 190 L/min). UAO resulted in lower peak inspiratory (25.2 ± 5.3 L/s, *P =* .01) and expiratory (33.6 ± 7.3 L/s, *P* = .03) flow than C (28.3 ± 10.0 and 38.0 ± 9.0 L/s). Longer inspiratory and expiratory durations in UAO resulted in lower breathing frequency (76.0 ± 16.0 vs. 89.2 ± 12.4 breaths/min). Post-exercise [lactate] was greater (*P* = .05) in UAO (6.8 [4.6-9.3] mmol/L) than C (3.5 [3.0-5.5] mmol/L). No evidence of EIPH was noted.

**Conclusions and clinical importance:**

Horses with UOA had marked changes in ventilation, however, $\dot{\rm V}$O_2_ and EIPH severity were not different to C, likely because the exercise was submaximal.

## Introduction

Upper airway obstructive respiratory tract conditions (UAO) are common causes of poor performance in sport horses, presumably due to their negative effects on ventilation. Athletic performance can be assessed by several means, including measuring an individual’s oxygen consumption ($\dot{\rm V}$O_2_) as a measure of pulmonary and cardiovascular function.^1^ Several studies have examined the effects of various UAOs on ventilation and $\dot{\rm V}$O_2._^2-6^ Exercise-induced pulmonary hemorrhage (EIPH) is another common disorder that can negatively affect performance^7^ and has been hypothesized to be exacerbated by UAO,^8^ although findings remain mixed.^9-11^ It is postulated that the severity of EIPH worsens when there is a rise in peak inspiratory (negative) pressures, as seen in diseases that lead to narrowing of the upper airway.^12^

Breed is considered an important risk factor for certain UAOs such as recurrent laryngeal neuropathy (RLN),^13^^,^^14^ but environmental factors, including rider intervention and head and neck positioning, are associated with conditions including laryngeal and pharyngeal instability.^15^ The Colombian Criollo horse is a unique breed that has smooth gaits which emphasize full collection, slow forward body movement, and high stride frequency. Abnormal respiratory noise is common in Colombian Criollo horses,^16^ presumably due to their tightly flexed head position, which exacerbates upper airway narrowing in other sport horses.^15^^,^^17^ Limited studies report the physiological workloads associated with the Colombian Criollo gaits,^18-22^ despite them serving as a useful model for other highly collected gait disciplines. Their movement, characterized by extreme collection and high mechanical stress, is associated with high heart rates but comparatively low $\dot{\rm V}$O_2_.^20^ Despite anecdotal reports of upper and lower airway disorders, including EIPH, there is only one study in Colombian Criollo horses that has examined upper respiratory function in horses presenting with abnormal respiratory noise, poor performance, or a combination of both.^16^ To our knowledge there is also only one study reporting on EIPH in competition-ready paso fino horses; however, this was based on post-exercise tracheobronchoscopy, which is considered to have lower sensitivity than bronchoalveolar lavage (BAL).^23^

It is unclear how obstructive upper airway conditions might affect performance in Colombian Criollo horses, which usually have vastly different workloads during training when compared to other sport horses.^20^^,^^21^ The objectives of this study were therefore to analyze the effects of UAO during exercise. The specific goals were to: (1) describe the association of upper airway dysfunction and $\dot{\rm V}$O_2_ and ventilatory variables, and (2) assess the effects of obstructive upper airway conditions on the prevalence and severity of EIPH. It was hypothesized that UAO would alter $\dot{\rm V}$O_2_ and spirometry variables, and result in more severe EIPH.

## Materials and methods

This was a cross-sectional observational study that took place in Antioquia, Colombia (altitude 1750 m). Animal care protocols were approved by the Veterinary Sciences Animal Care Committee of the University of Calgary. Twenty-three trained Colombian Criollo horses were recruited from the surrounding area using social media advertising and word of mouth. Recruitment was aimed at horses with abnormal respiratory noise, but control horses were also enrolled. Horses were classified as either healthy controls, or for having one or more of a variety of clinical signs of respiratory dysfunction, including cough, abnormal respiratory noise, sometimes combined with poor performance. All horses had been in regular training and were competition-ready. Horses were housed at one of two private farms (Farm 1 and Farm 2) in outdoor covered stalls and were fed a typical diet, comprised of Angleton hay, alfalfa pellets, a commercially available balanced feed and mineral salt. Water was provided ad libitum. Written owner consent was provided for all horses.

### Study protocol

On day 1, all horses underwent a physical exam by a veterinarian to ensure no overt injury or illness. Circumferential measurements estimated body weight.^24^ On day 2, an overground endoscopy (OGE) was performed during a standardized exercise test on all horses. A 1.5 m flexible endoscope (MEDView, MEDequus) was inserted into the horse’s left nostril without the use of a nose twitch and secured to a secondary noseband. The scope was advanced approximately 37-42 cm so that the larynx, tip of the epiglottis, and soft palate were visible within the frame. The endoscope was connected to a power unit secured on a saddle pad for recording and real-time video feedback was provided via wireless transmission to a portable screen. Horses were assessed at rest and during exercise, which consisted of a 10-min warm-up followed by a standardized exercise test (see details below). Videos were systematically reviewed (unblinded to horse identity) on a frame-by-frame basis by two experienced veterinary clinicians. Abnormal findings were classified using standardized nomenclature^25^ and grading systems.^26^^,^^27^

Horses were assigned to one of two groups based on findings from the baseline physical exam and OGE: (1) control (C): no clinical signs and no UAO; (2) UAO: UAO confirmed during exercise and the presence of one or more clinical signs (cough, respiratory noise, possibly combined with decreased performance).

Day 3 was a rest day and on day 4 the horses completed a standardized exercise test (SET) at their regular gait (either the trot, trocha, or paso fino) in a covered outdoor dirt arena. The arena at Farm 1 measured 12 m by 24 m with a 10 m wooden sounding board and that at Farm 2 was 12.5 m ×22 m with a 11 m wooden sounding board. Horses were ridden by one of two riders. After the 10-min warm-up, horses completed two laps around the arena, crossed the wooden sounding board, made an immediate turn, and returned across the board from the opposite direction. A final lap of the arena concluded the test.

Horses were equipped with a six-lead electrocardiograph to measure heart rate (HR) (INcardio Agile, INpulse Animal Health) and an ergospirometry facemask, previously validated for exercise^28^ and used to study Colombian Criollo gaits in healthy horses.^20^^,^^21^ Before the study, horses had been in regular training and had been acclimatized to wearing an un-instrumented facemask during exercise for at least 3 weeks. For the SET, the mask was fitted to each horse using adjustable straps and connected to a portable analyzer, secured in a backpack worn by the rider. Calibrations were performed before and after each exercise test using standardized medical grade gas (16.8% oxygen, 8.2% carbon dioxide, 75% nitrogen) and adjusted for environmental conditions, as previously described.^28^ Event markers identifying when the horses stepped onto and off the sounding boards were entered in real-time and cross-checked with video footage to ensure accurate timing for subsequent analysis. Horses in the UAO group also had the endoscope secured beneath the facemask during the SET for OGE recording. Exercise tests were stopped before completion in the event severe UAO resulted in obvious respiratory distress.

A bronchoalveolar lavage was performed 45 min after exercise using a described protocol^29^ to assess the occurrence and severity of EIPH. Briefly, after sedation and passage of a BAL tube with a cuff inflated in the wedged location, saline (250 mL) was infused into the lungs and then aspirated into syringes. Retrieved BAL fluid was transferred to tubes containing EDTA and colorimetrically assessed^30^ and then stored in a refrigerator pending cytological evaluation which occurred within 24 hours of finishing the exercise test. Two slides were prepared using at least 400 μL of BAL fluid each, centrifuged using a cytospin (Cytospin 4, Fischer Scientific) (90 g for 5 min) and stained (Hema-Tek 2000) using a modified Wright-Giemsa solution.^31^ The red blood cells (RBCs), erythrophages, and hemosiderophages were counted and reported in the cytological reports.

### Measured outcomes

Mean HR, $\dot{\rm V}$O_2_, and ventilatory variables (tidal volume, V_T_; peak inspiratory and expiratory airflows, Pk$\dot{\rm V}$_I_, Pk$\dot{\rm V}$_E_; inspiratory and expiratory time durations, t_I,_ t_E_; breathing frequency, f_b_; and minute ventilation, $\dot{\rm V}$E) were measured continuously throughout the SET and values were statistically analyzed during the segments when the horses crossed the wooden sounding boards since this was the highest intensity of exercise. Post-exercise blood samples were drawn from the right jugular vein as soon as horses stopped exercising, to measure blood lactate concentration ([La]) using a handheld analyzer (Lactate Plus; Nova Biomedical). The BAL fluid colorimetric assessment, RBC, and hemosiderophages’ counts were recorded. Exercise tests were videorecorded (1080p; iPhone 12, Apple) and stride frequency (strides/s) was calculated by dividing the total number of strides by the time (in seconds) it took to cross the boards. Videos were analyzed in slow motion (0.5× playing speed; QuickTime Player) on a large screen.

### Statistical analysis

Normality was assessed using the Shapiro–Wilk test, with descriptive data reported as mean ± SD if normally distributed or median [interquartile range (IQR)] otherwise. Comparisons between the C and UAO groups were made using unpaired *t*-tests with Welch’s correction and 95% CI of the differences were reported or a Mann–Whitney test when the distribution was not normal and actual difference was then reported. Significance was set to *P ≤* .05.

## Results

Twenty-three horses (5.0 [3.3, 7.0] years old; 352.2 ± 44.2 kg) completed the study. There were 16 mares, 2 geldings, and 5 stallions. Of these, 11 were designated as being in Group C, while 12 were assigned to the UAO Group. No relevant abnormalities were observed in any horse during the physical examination at rest. Ambient temperature, relative humidity, and barometric pressure during the SET were 26.7 °C [21.7, 27.9], 50% [45, 62] and 610 mmHg [609, 612], respectively.

### Baseline overground endoscopy

Twelve of the 23 horses (52%) had UAOs, with 6/12 having multiple diagnoses. Ten horses had RLN (Grade B, *n* = 5; Grade C, *n* = 5), with the left side affected in eight. One of the 10 had bilateral RLN (Grade-B). Nasopharyngeal collapse (NPC) was observed in 5/12 horses (Grade 1 = 3; Grade 3 = 2), medial deviation of the arytenoepiglottic fold (MDAF) (Grade 2) in 4/12, and vocal fold collapse (VFC) in 3/12 (Grade 2 = 2; Grade 3 = 1). Dorsal displacement of the soft palate (DDSP) was observed in one horse, which also had severe right-sided paralysis RLN and left arytenoid chondritis. Endoscopic findings are summarized in the Supplemental Material.

### Standardized exercise test

Twenty-two horses completed the full SET protocol which lasted 121 [110, 156] seconds. Sixteen horses (70%) were exercised at the trocha, 4 (17%) at the trot, and 3 (13%) at the paso fino gait. The test was terminated early in one horse due to presence of multiple UAOs, including DDSP, RLN, and left sided arytenoid chondritis, which resulted in severely limited airflow and exercise intolerance during the SET. This horse was removed from the analysis. The time to cross boards 1 and 2 were not different (*P* = .44) and took 6.2 [5.1, 7.5] seconds and 5.5 [4.3, 7.3] seconds, respectively. Due to the lack of difference, all variables were averaged between boards 1 and 2. Stride frequency was greater in the UAO Group than the C Group (2.8 ± 0.23, and 2.5 ± 0.27 strides/s, respectively; 95% CI, −0.52 to −0.07; *P* = .01); however, there was no difference in time to cross the boards (C: 5.6 [4.8, 6.7] s; UAO: 6.6 ± 1.3 s). Post-exercise [La] was greater in the UAO Group (C Group: 3.5 [3.0-5.5] mmol/L, UAO Group: 6.8 [4.6-9.3] mmol/L; Actual difference: 3.3; *P* = .05); however, no differences were observed in mean HR (Table 1).

**Table 1:** Relative and absolute oxygen consumption ($\dot{\rm V}$O_2_), ventilatory variables, and post-exercise blood lactate concentration measured in client-owned competition-ready Colombian Criollo horses during and after a submaximal standardized exercise performed at the trot, trocha, or paso fino gait, while wearing an ergospirometry facemask.

	**Control**	**UAO**
**Variable**	**(11)**	**(11)**
Heart rate (bpm)	140 ± 32	157 ± 33
$\dot{\rm V}$ O_2_ (ml/kg.min)	79.4 ± 22.0	88.2 ± 23.2
$\dot{\rm V}$ O_2_ (L/min)	28.9 ± 10.3	31.0 ± 10.0
f_b (_breaths/min)	89.2 ± 12.4	76.0 ± 16.0^*^
$\dot{\rm V}$ E (L/min)	723 ± 203	648 ± 190
Pk$\dot{\rm V}$E (L/s)	38.0 ± 9.0	33.6 ± 7.3^*^
Pk$\dot{\rm V}$I (L/s)	28.3 ± 10.0	25.2 ± 5.3^*^
Vt_E_ (L)	8.4 ± 2.0	8.6 ± 2.7
Vt_I_ (L)	7.9 ± 2.8	8.6 ± 2.1
t_E_ (s)	0.33 ± 0.04	0.37 [0.31, 0.46]^*^
t_I_ (s)	0.36 ± 0.05	0.43 ± 0.09^*^
t_E_:t_tot_	0.48 ± 0.02	0.47 ± 0.06
t_I_:t_tot_	0.52 ± 0.02	0.53 ± 0.06
Lactate (mmol/L)	3.5 [3.0, 5.5]	6.6 ± 3.2^*^

The exercise protocol consisted of a 10-min warm-up, followed by two laps of the arena, crossing a 10- or 11-m wooden sounding board, an immediate turn, and a return crossing in the opposite direction. Data analysis was completed while horses crossed the wooden sounding board. The test concluded with a final lap of the area. (Unpaired *t-*test for parametric data, Mann–Whitney for non-parametric data, ^*^P < 0.05).

Abbreviations: f_b_ = breathing frequency (breaths/min); Pk$\dot{\rm V}$E = peak expiratory flow (L/s); Pk$\dot{\rm V}$I = peak inspiratory flow (L/s); t_E_ = expiratory period (s); t_I_ = inspiratory period (s); t_E_:t_tot_ = expiratory ratio; t_I_:t_tot_ = inspiratory ratio; $\dot{\rm V}$E = minute ventilation (L/min); $\dot{\rm V}$O_2_ = oxygen consumption (ml/(kg.min)); Vt_E_ = expiratory tidal volume (L); Vt_I_ = inspiratory tidal volume (L).

**Figure 1:**
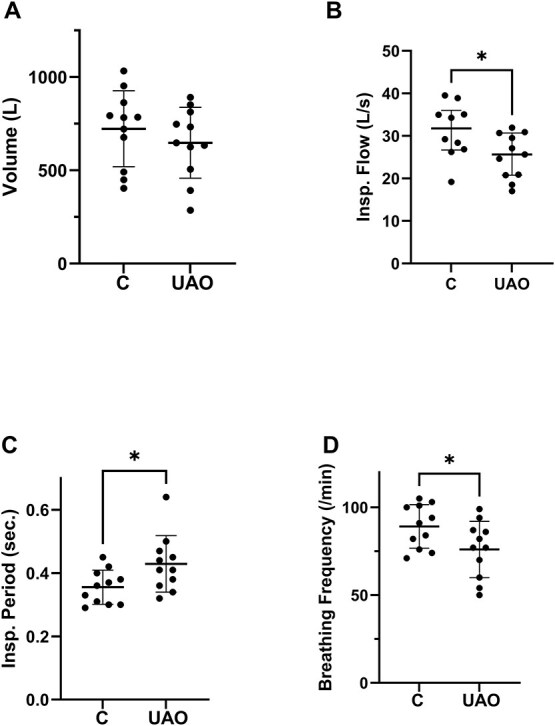
Ventilatory variables in 23 Colombian Criollo horses undergoing a standardized submaximal exercise field test while wearing an ergospirometry facemask. (A) Minute ventilation (L/min); (B) inspiratory flow (L/s); (C) inspiratory period (s); (D) breathing frequency (breaths/min) (^*^P < .05).

Oxygen consumption was not different between groups (C Group: 79 ± 22 mL/(kg min), UAO Group: 88 ± 23 mL/(kg min)), indicating a similar workload. With regard to spirometry, the UAO Group had lower Pk$\dot{\rm V}$I (25.2 ± 5.3 L/s; 95% CI, 1.05-14.6; *P* = .01) and Pk$\dot{\rm V}$E (33.6 ± 7.3 L/s; 95% CI, 0.6-11.4; *P* = .03) than the C Group (28.3 ± 10.0 L/s and 38.0 ± 9.0 L/s, respectively) but greater t_I_ (8.6 ± 2.1 s; 95% CI, 0.0-0.14; *P* = .03) and t_E_ (0.37 [0.31, 0.46] seconds; 95% CI, 0.0-0.17; *P* = .05) durations. This translated into a lower f_b_ in the UAO group (76.0 ± 16.0 breaths/min; 95% CI, −26.0 to −0.4; *P* = .04) than the C group (89.2 ± 12.4 breaths/min) (Figure 1). No statistical differences were detected between the other ventilatory variables, although comparisons were difficult due to irregular ventilatory patterns within the UAO group. Results for all ventilatory variables are shown in Table 1.

**Figure 2:**
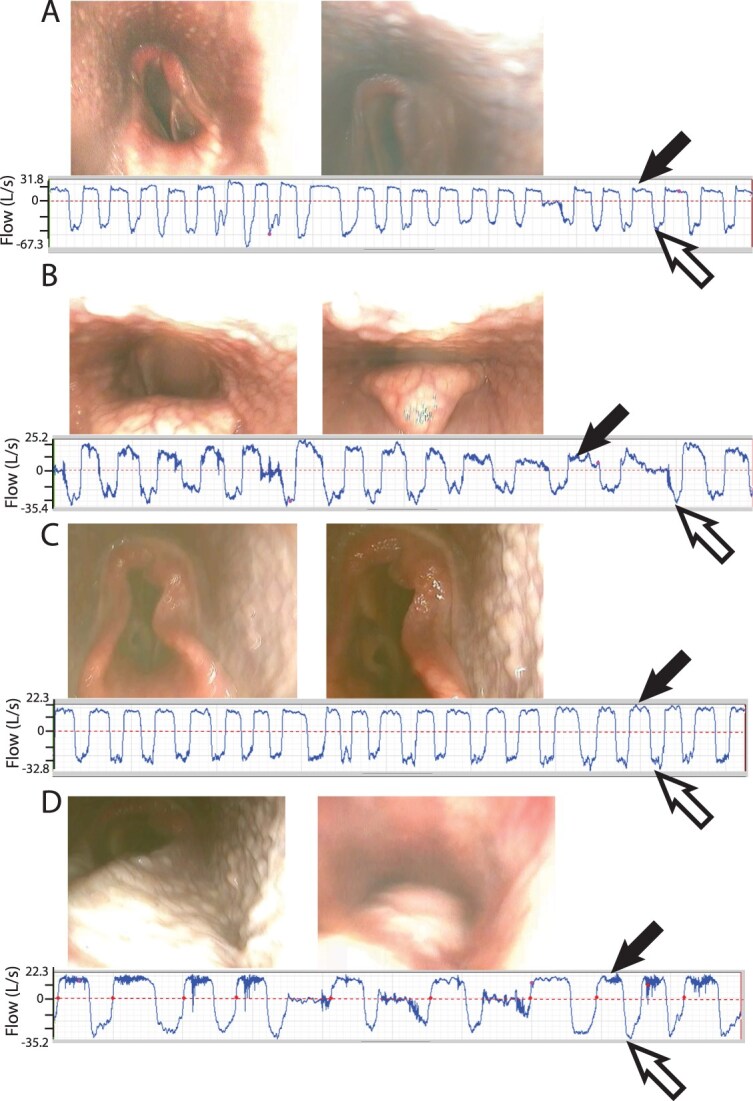
Examples of various upper airway conditions and the corresponding flow tracings (L/s) observed in Colombian Criollo horses during a standardized exercise test while wearing an overground endoscopy and ergospirometry facemask. Each tracing represents 15 s of exercise. The dotted line represents the zero line on the flow tracing, with inspiration shown as positive value and exhalation as negative values. The black arrows highlight some restricted inspiratory flows compared to the white arrows showing some greater expiratory flows in a few abnormal breaths. (A) Laryngeal hemiplegia (Grade-C); (B) nasopharyngeal collapse (Grade-1, dorsal collapse); (C) medial deviation of the arytenoepiglottic fold (Grade-3); and (D) dorsal displacement of the soft palate.

Examples of spirometry tracings are shown in Figure 2 where noticeable differences in f_b_ and V_T_ were observed among horses with different types of UAO. Recurrent laryngeal neuropathy, which primarily disrupts inspiration, was observed in 83% of horses with UAO. In addition to a distinct plateau in airflow during the inspiratory phase, prolonged inspiratory durations and increased t_I_:t_E_ were observed. Peak inspiratory flow was approximately half that of peak expiratory flow. These observations are shown in Figure 2A and were also accompanied by the typical “roaring” sound during inspiration. Nasopharyngeal collapse, shown in Figure 2B, was observed in 45% horses with UAOs, and was also associated with periods of severely decreased airflow. In the most extreme case, when complete dorsal pharyngeal collapse was present, airflow ceased entirely, as indicated by a flat line at 0 L/s on the flow trace. This could be heard as distinct pauses in breathing on video recordings. Other features of the spirometry analysis included artifact in the flow signal during the inspiratory phase of the horses with multiple UAOs present, when excess tissue (vocal folds and MDAF) were being pulled into the glottal opening and disrupting inspiratory flow. This is highlighted in Figure 2C. In the only horse with DDSP, the spirometry tracing showed a more erratic f_b_ along with a reduced Pk$\dot{\rm V}$E, possibly due to air being exhaled through the mouth, as shown in Figure 2D. The flow signal in this specific horse also demonstrated periods of no airflow when it was likely attempting to reposition the soft palate through active glottic closure. During video analysis, a loud gurgling noise could be heard on exhalation, when DDSP was present.

### Post-exercise BAL and assessment of EIPH

All 12 horses in the UAO group had post-exercise BAL but five horses in the C group did not undergo post-exercise BAL as they had an upcoming competition and did not consent to the required sedation. Post-exercise BALs showed an elevated neutrophil count (C Group: 16% [14-21], UAO Group: 19% [16-26]), which was indicative of a mild inflammatory response. Bronchoalveolar lavage cytology measurements were otherwise within normal limits.^32^ There was no evidence of EIPH as BAL fluid was confirmed to have “no color” in all horses,^30^ no erythrophagia was observed and RBC observation on BAL cytology was scarce. The greatest percentage of macrophages containing hemosiderin was only 1.8% and was observed in the UAO horses. A full description of post-exercise BAL is available in Table 2.

**Table 2:** Post-exercise bronchoalveolar lavage (BAL) cytology was collected from Colombian Criollo horses 45 min after completion of a standardized submaximal field exercise test.

	**C**	**UAO**
	(6)	(12)
Age (years)	4.5 [3.0, 8.0]	5.3 ± 1.8
Weight (kg)	359.1 ± 49.1	346.0 ± 40.3
Neutrophils (%)	17.7 ± 5.0	18.5 [16.0, 25.5]
Mast cells (%)	2.0 ± 0.6	1.7 ± 1.1
Eosinophils (%)	2.3 ± 1.4	2.0 [1.3, 4.0]
Macrophages (%)	72.2 ± 7.0	63.8 ± 13.6
Lymphocytes (%)	2.8 ± 0.8	3.0 [2.0, 4.0]
BAL color	No color (*n* = 6)	No color (*n* = 12)
RBC observation on BAL cytology	None (*n* = 5) Scarce (*n* = 1)	None (*n* = 7) Scarce (n = 5)
Erythrophage (%) Hemosiderophage (%)	0.0 [0.0, 0.0] 0.0 [0.0, 1.3]	0.0 [0.0, 0.0] 0.5 [0, 1.8]

Abbreviations: BAL = bronchoalveolar lavage; RBC = red blood cells.

Horses in the control group (C) (*n* = 6) had no clinical signs and no evidence of upper airway obstruction (UAO), as confirmed with overground endoscopy during exercise. Horses in the UAO group (*n* = 12) exhibited one or more upper airway obstructions during exercise and showed associated clinical signs, including respiratory noise, coughing, and/or decreased or poor performance.

Supplemental Material.

## Discussion

Increased or abnormal respiratory noise is common in Colombian Criollo horses and was present in all horses within the UAO group during the SET. This was presumably due to their tightly flexed head position, which exacerbates obstructive airway diseases in sport horses.^15^^,^^17^^,^^33^ More than 87% of Colombian Criollo horses presenting with abnormal noise combined or not with poor performance have arytenoid cartilage collapse.^16^ Our study found a similar prevalence (83%). Severity of RLN increased with exercise intensity, which is hypothesized to result from greater poll flexion, higher negative pressures associated with efforts to generate higher flow rates through narrower upper airway lumens.^16^ Laryngeal hemiplegia results in lower $\dot{\rm V}$O_2_ and significantly higher inspiratory impedance and airway pressures in thoroughbreds exercising maximally on a treadmill.^2^^,^^3^^,^^34^ However, as found in our study, $\dot{\rm V}$O_2_ was not different between horses with or without RLN during submaximal workloads.^35^ The trot, trocha, and paso fino gait of the Colombina Criollo horse are characterized as submaximal exercise.^20^^,^^21^

In contrast to RLN, DDSP primarily resulted in expiratory obstruction. $\dot{\rm V}$O_2_ has been shown to decrease by 10% and $\dot{\rm V}$E by 13% when DDSP is present, primarily due to a reduction in Vt but not f_b_.^5^ In the current study, there was only one horse with DDSP which persisted for approximately 30 s when the test was terminated. This horse had a lower Pk$\dot{\rm V}$E, and lower Vt and $\dot{\rm V}$E, which could potentially be attributed to air being diverted through the mouth^36^; however, this horse also had right sided RLN (Grade-IV) and severe chondritis of the left arytenoid, which likely had a negative effect on ventilatory variables. Results from studies examining $\dot{\rm V}$O_2_ and ventilatory variables during exercise with other UAOs are limited. Pharyngitis and VFC do not result in any significant differences in $\dot{\rm V}$O_2_ in maximally exercising horses,^2^ though blood gases are worse in horses with severe pharyngeal lymphoid hyperplasia, which might be attributed to lower respiratory disease.^37^ Horses with palatal dysfunction and DDSP^5^^,^^6^ have lower $\dot{\rm V}$E, Vt, $\dot{\rm V}$O_2_ (but not f_b_) but significant decreases were only observed with DDSP.

No differences were observed in $\dot{\rm V}$E or Vt between UAO and C, but horses in the UAO group had a lower f_b_, presumably due to the longer inspiratory and expiratory durations associated with obstruction. While our findings suggest that UOAs did not lower $\dot{\rm V}$O_2_ during submaximal exercise, it remains unclear whether there were differences in relative workloads between groups as peak $\dot{\rm V}$O_2_ was not measured (maximal intensity gallop is not attainable in these horses) and it is conceivable that the UAO horses had a lower peak $\dot{\rm V}$O_2_ than the C horses. Combined with a post-exercise [La] that was higher in horses in the UAO group than controls, our findings suggest that a greater relative workload was likely in the UAO horses. The greater post-exercise [La] was a finding consistent with a previous study examining RLN in racehorses^4^ which found that higher grades were associated with greater lactate concentrations. Although arterial gases were not measured in our study, horses with UAO are more hypoxemic that control horses, even at low speeds,^38^ resulting in an earlier transition to anaerobic metabolism and earlier blood lactate accumulation. Horses in the UAO group also had a greater stride frequency; however, no differences in time to complete the board could indicate horses were taking shorter strides. The relationship between stride frequency and breathing frequency was disrupted in UAO horses, since horses had a greater fs:fb due to having a greater fs with a lower fb than the C horses. However, the lack of difference in mean HR and time to cross the board suggests that the higher [La] might have been due to the reduced alveolar ventilation and oxygen delivery to the locomotory muscles. Measuring peak $\dot{\rm V}$O_2_ and the associated heart rate response is, however, challenging in this breed of horses. Regardless, the submaximal nature of the trocha exercise enabled most horses with UAO to ventilate sufficiently well to complete the exercise test in the same times as the C group.

Distinct spirometry patterns were observed across specific UAOs, but these were purely descriptive as sample sizes were insufficient to perform meaningful statistical analysis comparing spirometric measurements associated with different causes of UAO.

All horses had elevated proportion of BAL neutrophils after exercise (>5%) without evidence of respiratory clinical signs in the baseline examination at rest. Intense exercise increases the BAL neutrophil response in horses,^39^^,^^40^ which might have contributed to this observation, even though the exercise performed in this study was of submaximal intensity. The mildly elevated proportion of neutrophils in the current study might also suggest a mild inflammatory response,^32^ which was possibly due to environmental exposure to dust, allergens and lipopolysaccharides associated with being introduced to new outdoor stalls shortly before beginning the study. Horses might have also had an elevated neutrophilic response due to transport,^41^ as well as new environmental exposures associated with study location.

There are anecdotal reports of EIPH in Colombian horse breeds. A recent study investigating the prevalence in healthy horses performing the Paso Fino gait found that 27% of horses had EIPH (endoscopic Grade 1).^20^ A higher percentage may have been observed if BALs had been performed in that study, as they have greater test sensitivity than endoscopic score alone.^23^ In the current study horses showed no signs of EIPH, based on negative colorimetric assessment.^30^ The submaximal nature of the exercise was likely responsible for the lack of EIPH.

### Limitations

Although 23 horses were recruited to participate in the study, the sample size remained small and might be subject to a selection bias of cases. Additionally, the variations in UAO diagnosis made it difficult to make meaningful comparisons as each UAO diagnosis had unique ventilatory characteristics. Horses were not ridden by the regular rider and might not have been exercised at the same intensity as when usually trained. Differences in individual training status may have also affected results. Due to the nature of the training and exercise, we were unable to measure peak $\dot{\rm V}$O_2_ and thus were unable to determine the %$\dot{\rm V}$O2max at which the horses were exercising. Anecdotal reports of EIPH may also have been associated with longer training sessions. In addition, hemocytometry was not performed and although we could assess the color of the BAL we are unable to truly assess the extent of EIPH without hemocytometry and post-exercise endoscopy.

### Conclusion

$\dot{\rm V}$ O_2_ and spirometry were simultaneously reported during this study field test in horses with UAOs. Laryngeal hemiplegia and nasopharyngeal collapse were the most prevalent UAO in horses exhibiting one or more clinical signs of UAO (abnormal respiratory noise, cough, with or without decreased performance) in this selected cohort. Breath-by-breath analysis revealed marked differences in ventilation, particularly in peak inspiratory and expiratory flows and durations, resulting in lower respiratory rates in those with UAO when compared to control horses. However, the exercise intensity of the trocha gait was likely not sufficient to negatively affect $\dot{\rm V}$O_2_ or reduce performance_._ Horses appeared to be able to effectively compensate for the ventilatory restrictions caused by the obstructions. The lack of EIPH was likely due to the submaximal nature of the exercise.

## Supplementary Material

supplementary_material_aalag111

## Off-label antimicrobial declaration

Authors declare no off-label use of antimicrobials.

## Institutional animal care and use committee or other approval declaration

Institutional Animal Care and Use Committee approval provided by Institución Universitaria Visión de las Américas.

## Abbreviations

BAL: bronchoalveolar lavage
C: control
DDSP: dorsal displacement of the soft palate
EIPH: exercise-induced pulmonary hemorrhage
fb: breathing frequency
HR: heart rate
LH: laryngeal hemiplegia
MDAF: medial deviation of the arytenoepiglottic fold
OGE: overground endoscopy
Pk$\dot{\rm V}$E: peak expiratory flow
Pk$\dot{\rm V}$I: peak inspiratory flow
RBC: red blood cells
RLN: recurrent laryngeal neuropathy
SET: standardized exercise test
tE: expiratory time duration
tI: inspiratory time duration
UAO: upper airway obstruction
$\dot{\rm V}$ E: minute ventilation
VT: tidal volume
VFC: vocal fold collapse
$\dot{\rm V}$ O2: oxygen consumption
[La]: blood lactate concentration
