# Supplementary material for: The effects of upper airway obstruction on oxygen consumption and ventilatory variables in Colombian criollo horses
Source: J Vet Intern Med. 2026 Jun 17;40(3):aalag111. doi: 10.1093/jvimsj/aalag111 (PMC13275017; doi:10.1093/jvimsj/aalag111)
Supplement: supplementary_material_aalag111 [file supplementary_material_aalag111.docx]

**Supplementary Material**

Type and severity of upper airway obstruction (UAO) observed in 23 client-owned, competition-ready Colombian Criollo horses during a submaximal standardized exercise test. Horses were fitted with a 1.5-m flexible endoscope (MEDView, MEDequus) secured to a customized saddle pad and were assessed at rest, and during exercise. The exercise protocol included a 10-minute warm-up, followed by two laps of the arena, crossing a 10- or 11-m wooden sounding board, an immediate turn, and a return crossing in the opposite direction. The test concluded with a final lap of the area. Overground endoscopy recordings were systematically reviewed and graded^25,26^ on a frame-by-frame basis by two experienced clinicians.

Briefly, RLN was graded using a Grade A-D grading system (Rossignol et al., 2018). All other UAO diagnoses were scored in accordance with grading systems compiled in supplementary material provided by McGivney et al., (2017). NPC was graded using a 0-4-point grading system, VFC graded using a 3-point grading system, MDAF graded using a 0–3-point grading system, and PI using a 0–3-point grading system.

|  | **UAO** | **Grade** |
| --- | --- | --- |
| Horse 1 | RLN | Grade-B |
| Horse 2 | RLN | Grade-C |
| Horse 3 | RLN + NPC | Grade-C, Grade 1 |
| Horse 4 | RLN | Grade-C |
| Horse 5 | NPC | Grade 3 |
| Horse 6 | RLN + VFC + MDAF | Grade-B, Grade 2, Grade 2 |
| Horse 7 | RLN + VFC + MDAF + NPC | Grade-B (R/L), Grade 2, Grade 2, Grade 3 |
| Horse 8 | RLN + iDDSP + Arytenoid Chondritis | Grade-IV (R), Left side |
| Horse 9 | RLN + MDAF | Grade-C, Grade 2 |
| Horse 10 | NPC | Grade 1 |
| Horse 11 | RLN | Grade-B |
| Horse 12 | RLN + NPC + VFC + MDAF | Grade-B (R), Grade 1, Grade 3, Grade 2 |

RLN: recurrently laryngeal hemiplegia; NPC: nasopharyngeal collapse; VFC: vocal fold collapse; iDDSP: intermittent dorsal displacement of the soft palate; MDAF: medial deviation of the aryepiglottic folds; L: left side; R: Right side; R/L: right and left sided.
